# Development of synthetic anti-cyclic citrullinated peptide antibody and its arthritogenic role

**DOI:** 10.1038/cti.2015.24

**Published:** 2015-11-27

**Authors:** Youngkyun Kim, Jennifer Lee, Hyerin Jung, Hyoju Yi, Yeri Alice Rim, Seung Min Jung, Ji Hyeon Ju

**Affiliations:** 1 CiSTEM Laboratory, Convergent Research Consortium for Immunologic Disease, College of Medicine, Seoul St Mary's Hospital, The Catholic University of Korea, Seoul, Republic of Korea; 2 Division of Rheumatology, Department of Internal Medicine, College of Medicine, The Catholic University of Korea, Seoul, Republic of Korea

## Abstract

This study was undertaken to develop a novel anti-citrullinated peptide antibody (ACPA) and to investigate its arthritogenicity in a collagen-induced arthritis (CIA) model. The novel ACPA, 12G1, was developed by injecting cyclic citrullinated antigen in mice and subsequently hybridizing the B cells producing citrullinated peptide-specific antibodies with a myeloma cell line. The arthritic joints of mice with CIA and collagen antibody-induced arthritis (CAIA) as well as interleukin-1 receptor antagonist (IL-1Ra) knockout (KO) mice were stained immunohistochemically using the 12G1 antibody. Confocal immunostaining was used to identify colocalization of 12G1 with various citrullinated proteins. 12G1 in the presence or absence of chelating beads was administered to CIA mice on days 21 and 28 after type II collagen (CII) immunization to investigate 12G1 arthritogenecity. 12G1 detected citrullinated proteins in the arthritic joints of all the experimental arthritis models used. Confocal immunostaining showed that 12G1 was colocalized with well-known citrullinated proteins, including vimentin, collagen, anti-immunoglobulin binding protein and fibronectin. Staining of citrullinated proteins using 12G1 was more diffuse in CIA mice compared with CAIA and IL-1Ra KO mice. 12G1 injection apparently acted as a booster of immunization in CIA mice in combination with a single CII immunization, with this effect being abolished when 12G1 was injected with chelating beads. The novel ACPA, 12G1, identified various citrullinated proteins in the arthritic joints of three experimental arthritis models. 12G1-treated mice developed arthritis following a single CII immunization, suggesting an arthritogenic potential for ACPA in CIA mice.

Rheumatoid arthritis (RA) is a systemic autoimmune disease characterized by chronic joint inflammation that can lead to cartilage loss and bone erosion.^1^ As implied by the term ‘autoimmune', autoantibodies are found in the sera of RA patients. In addition to classical autoantibody ‘rheumatoid factor', anti-citrullinated peptide antibodies (ACPAs) are involved in the disease and have high diagnostic and predictive value.^2, 3^ ACPA is more specific for RA than rheumatoid factor, and is associated with the more severe disease phenotype of more frequent extra-articular manifestation^4^ and joint destruction.^5^

Peptide citrullination is a physiologic process, whereby peptidyl arginine deiminase converts s-peptidyl arginine into a peptidyl citrulline.^6^ Although citrullination commonly occurs in inflammatory conditions and is therefore not specific to RA,^7^ citrullinated proteins are found abundantly in RA arthritic joints, whereas they are rarely detected in healthy joints.^8^ In addition, citrullinated fibrin is found in the murine model of collagen-induced arthritis (CIA) and streptococcal cell wall-induced arthritis.^9^ Several researchers considered these citrullinated proteins as autoantigens in RA and investigated whether they contributed to autoimmune arthritis development in animal models. Indeed, autoimmune arthritis was induced by administrating citrullinated type II collagen (CII) in the absence of adjuvant,^10^ whereas immunization using citrullinated fibrinogen led to inflammatory arthritis in HLA-DR4 transgenic mice.^11^ Citrullinated proteins known to be associated with RA include fibrin,^12^ vimentin,^13^ fibronectin,^8^ anti-immunoglobulin binding protein (BiP)^14^ and CII.^15^

The antibody against these citrullinated proteins—ACPA—is detected in the sera of RA patients many years before clinically overt arthritis is present, indicating that ACPA may play an important role in RA pathogenesis.^16^ However, it remains unclear whether ACPA plays a causative, pathogenic role in RA pathogenesis or whether it is simply a bystander, resulting from joint inflammation. Although many researchers have investigated this issue, conflicting data were reported according to the different experimental materials and methods.^7, 15, 17, 18^

Here, we developed a novel citrulline-specific monoclonal antibody that could detect citrullinated proteins in arthritic joints and investigated whether there were any differences in the expression patterns of citrullinated proteins according to the experimental arthritis model. Furthermore, we addressed the issue of the arthritogenic potential of ACPA using our novel ACPA, termed 12G1 antibody, in a CIA model.

## RESULTS

### Development of a novel antibody against citrullinated peptide, 12G1

**Figure 1:**
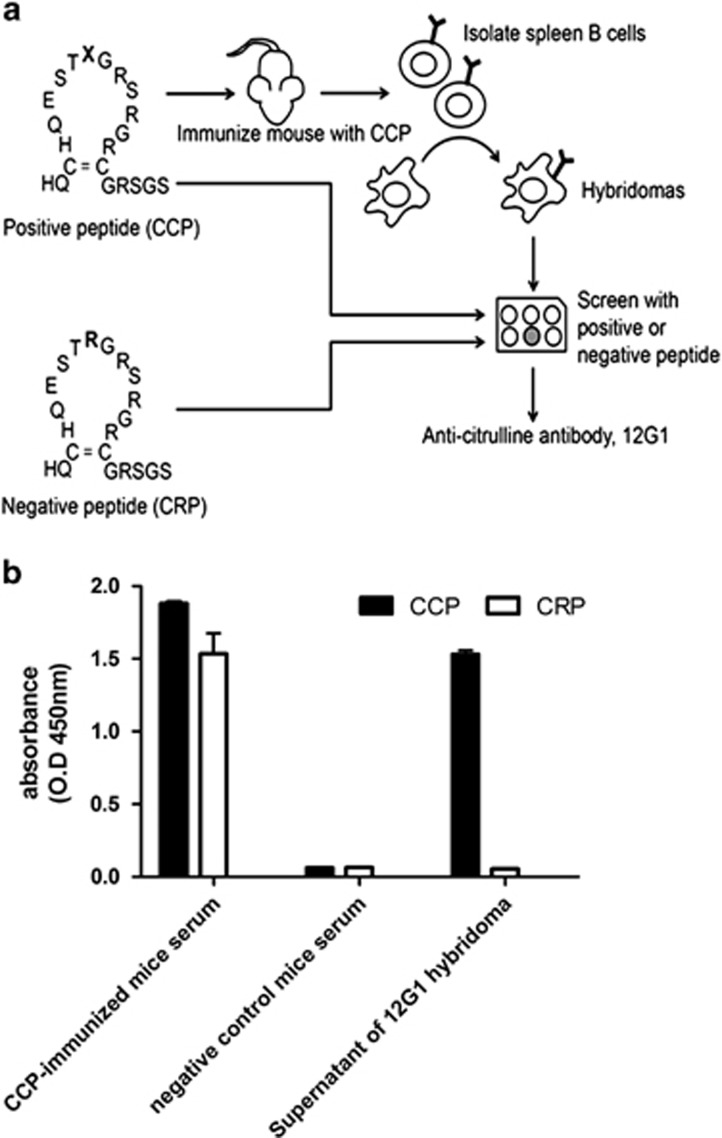
Generation of the citrulline-specific mAb, 12G1. (**a**) A schematic diagram of 12G1 generation. (**b**) Sera from nonimmunized and immunized mice and the supernatant from the hybridoma cells underwent ELISA to detect CCP or CRP. 12G1 specifically detected CCP.

The process of generating the novel antibody 12G1 to cyclic citrullinated peptide (CCP) is presented in Figure 1a. A previously reported cyclic-structured synthetic peptide, which included a citrullinated filaggrin subunit, was used as the antigen to generate a monoclonal antibody (mAb) to CCP.^19^ Four mice were immunized using this synthetic peptide. The mouse with antibodies that displayed the highest affinity for CCP and the weakest binding to the control peptide, cyclic arginine peptide (CRP), which contained arginine instead of citrulline, was selected. B cells obtained from this mouse were fused with a myeloma cell line to generate a hybridoma cell line that produced mAbs. To identify the correct clone producing anti-CCP-specific mAb, enzyme-linked immunosorbent assay (ELISA) was performed using CCP and CRP, respectively. This process was repeated until we isolated a single clone (designated as 12G1) that secreted a mAb with high affinity for CCP, but not for CRP. As shown in Figure 1b, we confirmed that 12G1 reacted specifically with CCP, whereas the sera of the CCP-immunized mice reacted with both CCP and CRP. The sera of nonimmunized mice (negative control) did not appear to detect CCP or CRP.

### 12G1 detected the citrullinated proteins in various experimental arthritis models

**Figure 2:**
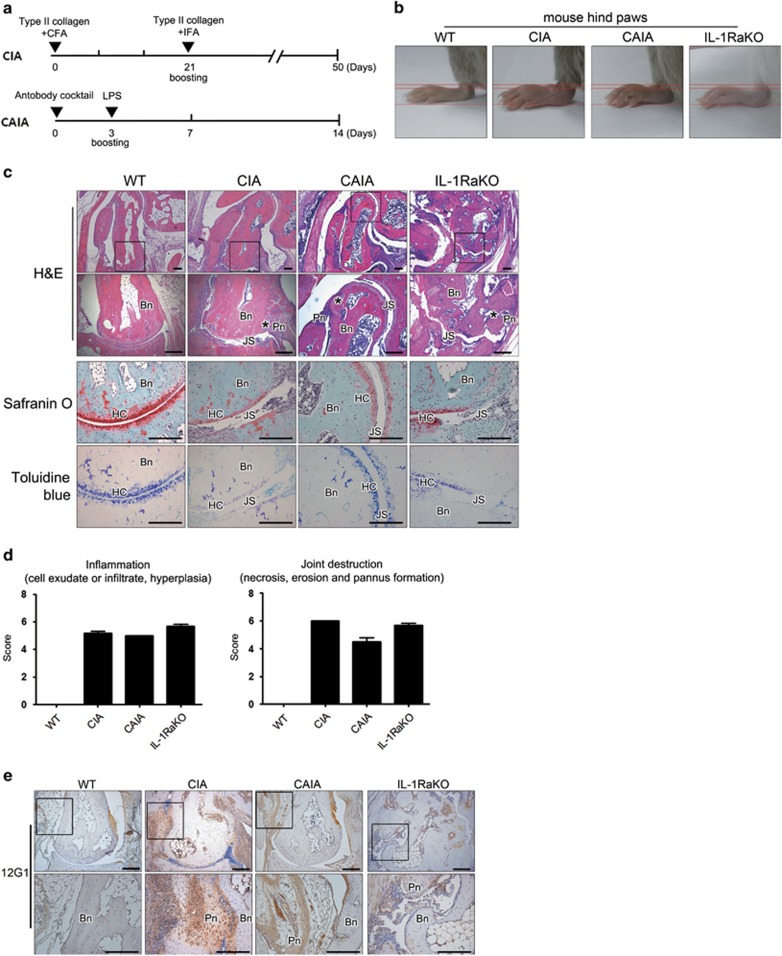
12G1 detected citrullinated peptides in the arthritic joints of various experimental arthritis models. (**a**) A schematic diagram of CIA and CAIA induction in DBA1/J mice. (**b**) Representative hind paws of each arthritis model (CIA: on day 50 after first immunization; CAIA: on day 14 after first immunization; IL-1Ra KO: 4 weeks old). (**c**) Representative histological features of the joints of each arthritis model. The results of hematoxylin and eosin (figures of the lower panel are the magnifications of the highlighted areas of the upper panel), Safranin O and Toluidine Blue staining. Bn, bone; HC, hyaline cartilage; JS, joint space; Pn, inflamed pannus. Asterisk denotes cartilage and bone loss. (**d**) Semiquantitative scores for histologic findings in terms of inflammation (left) and joint destruction (right). Three independent observers graded in a blinded manner. (**e**) Arthritic joints of each arthritis model were immunostained for citrullinated peptides using 12G1. Figures of the lower panel are the magnifications of the highlighted areas of the upper panel.

We investigated whether 12G1 could detect citrullinated proteins in the joints of the experimental mouse arthritis models CIA, collagen antibody-induced arthritis (CAIA) and interleukin-1 receptor antagonist (IL-1Ra) knockout (KO) (Figure 2a). The joints with overt arthritis of each experimental model (Figure 2b) underwent histologic evaluation. As shown in Figures 2c, the representative arthritic joint of each model displayed inflammatory cell infiltration and joint destruction. Pannus formation and bone destruction appeared more severe in CIA and IL-1Ra KO mice compared with CAIA mice. Tarsal bone destruction was most prominent in IL-1Ra KO mice. However, there was no statistically significant difference in the inflammation or joint-destruction scores among the representative joints of the various models (Figures 2d). When arthritic joints were stained using 12G1 mAb, citrullinated proteins were detected in the synovium of all three experimental models (Figure 2e). Citrullinated proteins were detected more prominently in the CIA mice compared with the CAIA and IL-1Ra KO mice. Most of the citrullinated proteins were observed in the region of the inflamed pannus adjacent to the bone.

### Comparison of citrullinated protein expression detected using 12G1 in various experimental arthritis models

**Figure 3:**
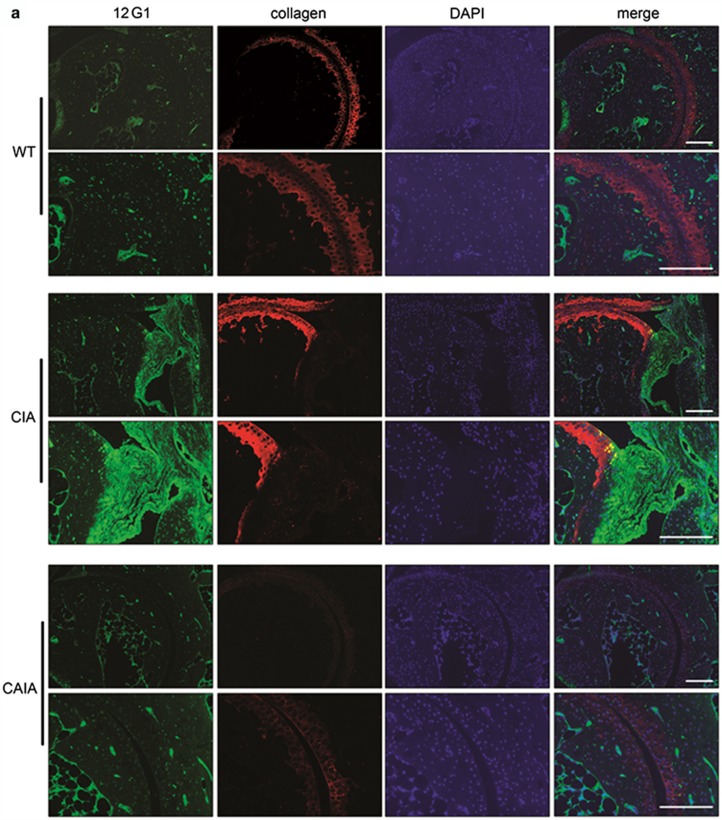

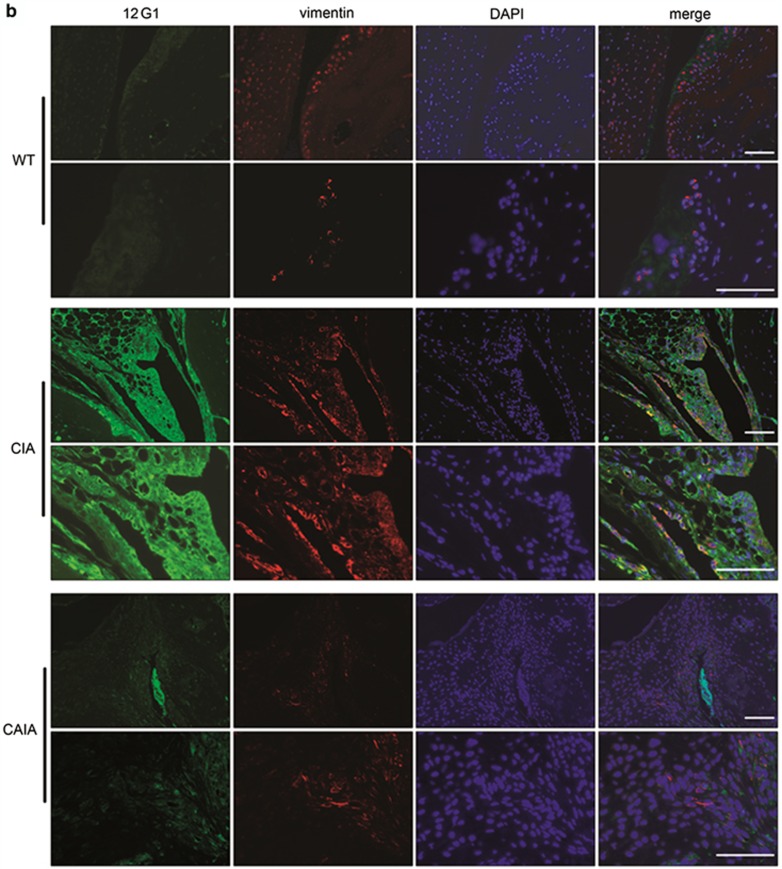

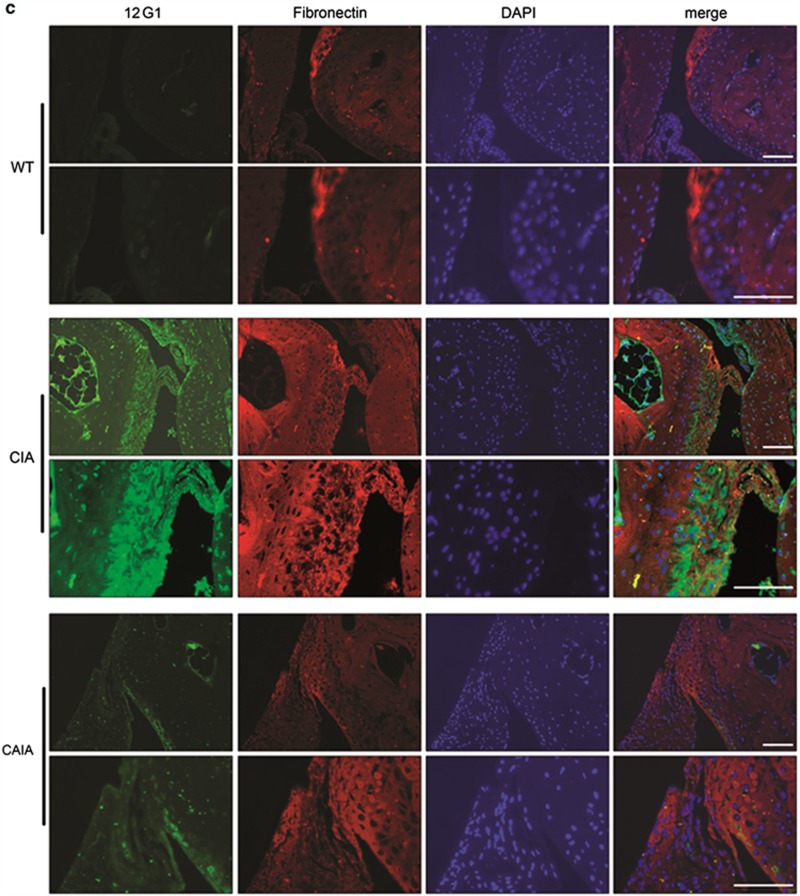

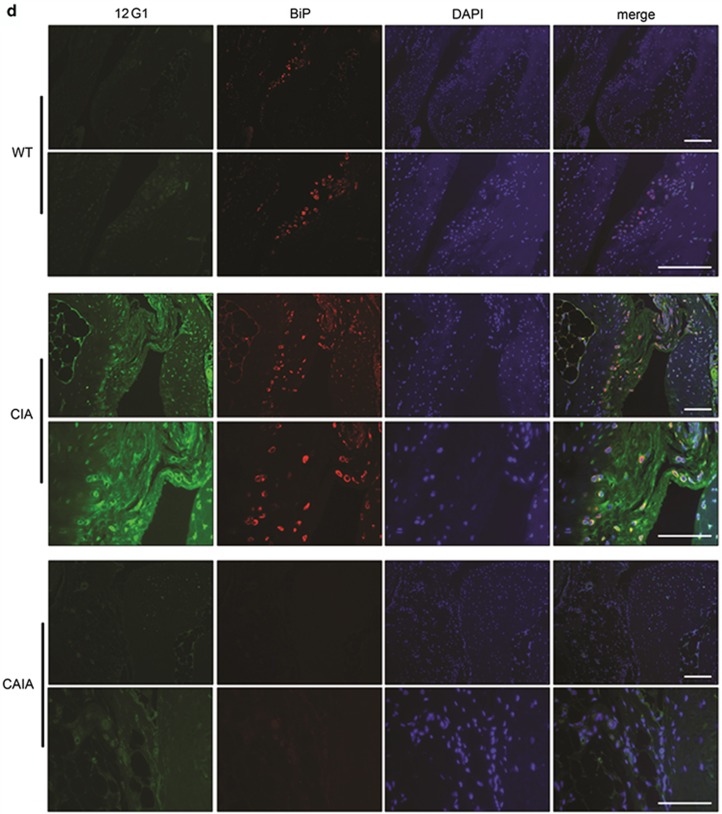
12G1 identified RA-associated citrullinated proteins in the arthritic joints of experimental arthritis models. Hind paws of each arthritis model were subject to confocal immunostaining for (**a**) collagen, (**b**) vimentin, (**c**) fibronectin and (**d**) anti-immunoglobulin binding protein. 4′,6-Diamidino-2-phenylindole staining was performed to investigate whether the citrullinated proteins were located intracellularly or extracellularly. Protein expression was quantified based on staining intensity.

We attempted to identify the citrullinated proteins to which 12G1 was bound. The representative citrullinated proteins in RA have been reported to include vimentin,^8^ collagen,^20^ BiP^14^ and fibronectin.^21^ Therefore, we investigated whether 12G1 could identify these citrullinated proteins in the arthritic joints of various experimental arthritic models. Fluorescence immunostaining of joint sections demonstrated that all four citrullinated proteins could be detected using 12G1 (Figures 3a–d). Consistent with the immunohistochemistry findings, the citrullinated proteins were more abundant in CIA mice compared with the other two models, and most of the citrullinated proteins were detected near the inflamed pannus. Of note, the location of citrullinated protein in the joint differed according to the characteristic of each protein. As expected, citrullinated collagen was more frequently observed in cartilage, of which CII collagen is the major component (Figure 3a). In contrast to the other proteins located in the extracellular stroma, citrullinated BiP was identified in the cytoplasm (Figure 3d), possibly because it is an endoplasmic reticulum protein.

### CIA was induced using a single immunization without booster when 12G1 was additionally injected

**Figure 4:**
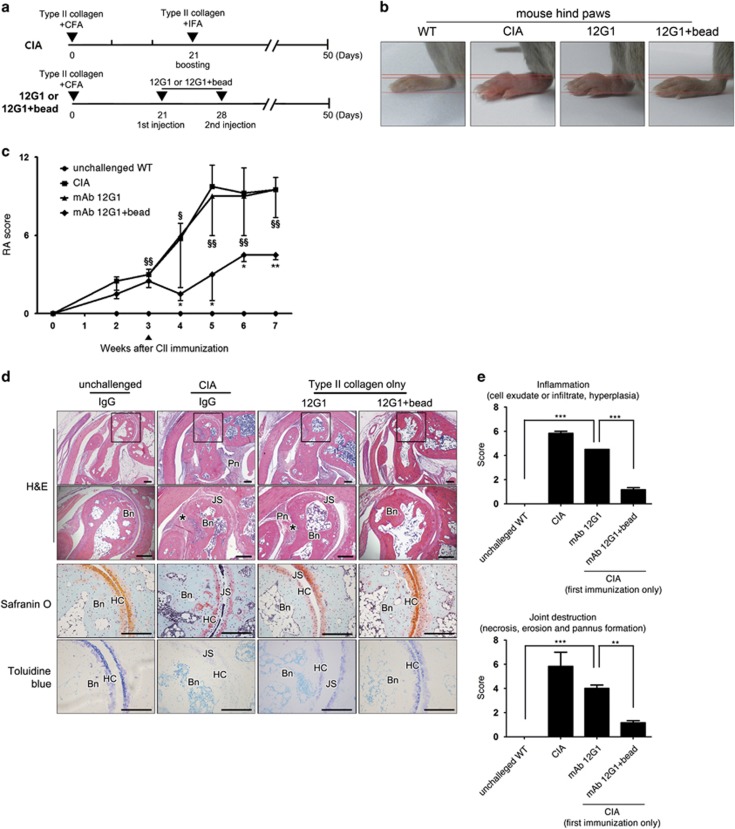
CIA was induced using a single immunization without booster when 12G1 was additionally injected. (**a**) A schematic diagram of 12G1-induced arthritis. 12G1 or 12G1 with chelating beads was administered on days 21 and 28 after the first immunization with CII. (**b**) A representative hind paw of wild-type (WT), CIA, 12G1-treated and 12G1 with bead-treated mice on day 50 after the first immunization. (**c**) Clinical arthritis scores for each group during the experimental period. A triangle denotes the time for the second booster for the CIA group, and the first 12G1 with/without bead administration for the 12G1 group. *12G1-treated versus 12G1 with beads-treated group; ^§^12G1-treated versus unchallenged WT. (**d**) Representative histological features of the joints of each arthritis model. The results of hematoxylin and eosin (figures of the lower panel are the magnifications of the highlighted areas of the upper panel), Safranin O and Toluidine Blue staining. Bn, bone; HC, hyaline cartilage; JS, joint space; Pn, inflamed pannus. (**e**) Semiquantitative scores from the histologic findings in terms of inflammation (upper) and joint destruction (lower). Three independent observers graded in a blinded manner. *^,§^*P*<0.05; **^, §§^*P*<0.01; ***P<0.001.

**Figure 5:**
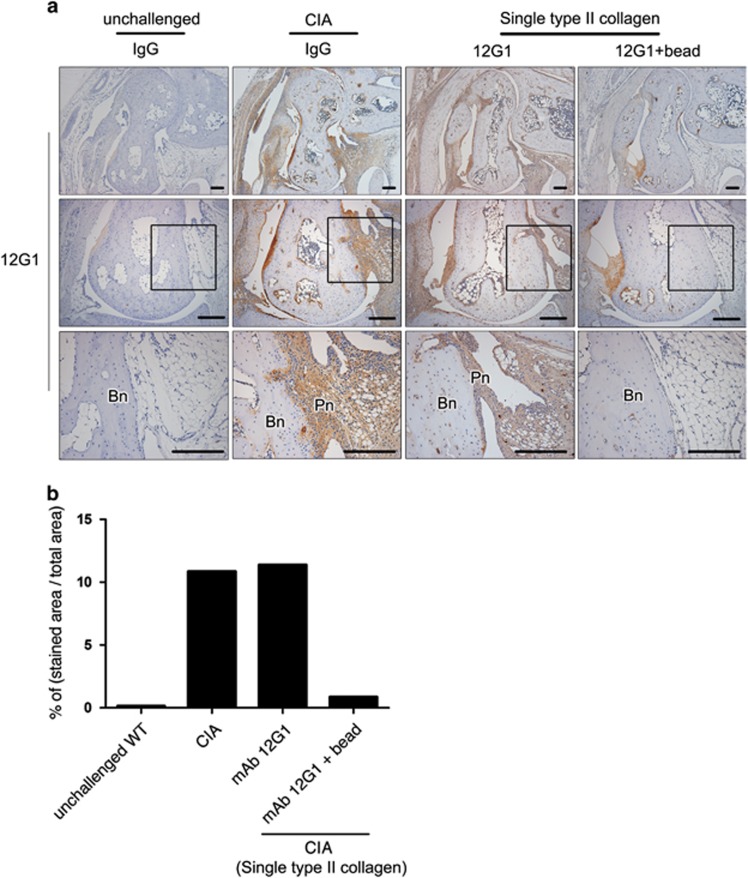
Citrullinated peptides were detected by 12G1 in the arthritic joints of 12G1-induced arthritis mice. (**a**) Immunohistochemical staining for citrullinated peptides using 12G1 in the joints of wild-type, CIA, 12G1-treated and 12G1 with beads-treated mice. Figures of the bottom panel are the magnifications of the highlighted areas of the figures of the middle panel. Bn, bone; Pn, inflamed pannus. (**b**) Ratio of the positively stained area to the total area of the immunohistochemistry data from (**a**).

As previous studies reported conflicting results regarding the arthritogenicity of ACPA,^15, 17^ we investigated whether 12G1 plays a pathogenic role in CIA development (Figure 4a). Interestingly, the mice treated using 12G1, instead of a booster, on days 21 and 28 after a single CII immunization displayed a comparable arthritis incidence and severity to those of conventional (with booster immunization) CIA mice (Figures 4b and c). Notably, this effect was completely abolished when 12G1 was injected in combination with chelating beads. Histological evaluation consistently demonstrated that the arthritic joints of 12G1-injected CIA mice displayed a similar level of inflammation and joint destruction as CIA mice, whereas those of the 12G1 plus beads-treated group showed a lesser degree of inflammation and joint destruction (Figures 4d and e). These data suggest that 12G1 could act at least as a booster, although its solitary role could not be demonstrated in this setting. Citrullinated proteins were detected using 12G1 in conventional CIA mice and 12G1-treated CIA mice, whereas 12G1-bound citrullinated proteins were not observed in the joints of mice treated using 12G1 plus beads (Figures 5a and b).

## DISCUSSION

Here, we reported the generation of a novel ACPA that could detect various citrullinated proteins in arthritic joints. Immunostaining of arthritic joints using our novel 12G1 demonstrated that it was colocalized with well-known citrullinated proteins in RA, including vimentin, collagen, BiP and fibronectin. Citrullinated protein-bound 12G1 was most abundant in the arthritic joints of CIA mice among the three experimental arthritis models investigated. Although 12G1 injection alone was unable to induce arthritis, it did appear to have an effect comparable to a booster immunization in CIA mice. Moreover, the fact that the deprivation of 12G1 by chelating beads abolished the arthritogenic effect suggests the pathogenic role of 12G1 in CIA, an experimental RA model.

12G1 identified various citrullinated proteins, regardless of how the arthritis was induced. We observed that citrullinated proteins were also present in the arthritic joints of the IL-1Ra KO mice model, although to a lesser extent, confirming that citrullinated proteins were not restricted to the CIA or CAIA models. Therefore, the roles of citrullinated proteins and ACPA were not determined by the mechanism of arthritis development. It appears that following development of synovial inflammation, protein citrullination is induced regardless of the induction method. These findings are also consistent with the fact that citrullination is not specific for RA, but also occurs in an inflammatory condition.^22^

The main aim of our study was to determine whether ACPA could elicit arthritis in RA models, because previous studies reported conflicting results. Kuhn *et al.*^17^ reported that anti-citrullinated fibrinogen antibody alone did not induce arthritis; rather, it enhanced arthritis when coadministered with a submaximal dose of anti-CII antibody. In contrast, Uysal *et al.*^15^ demonstrated that anti-citrullinated collagen antibody injection was sufficient to induce arthritis in a susceptible strain, although the severity of the arthritis induced varied according to the epitope recognized by a given Ab. The discrepancy between these studies has been explained by the differences in mice strain and experimental methods used. In the present study, we used 12G1 as a booster in DBA1/J mice with CIA. Cantaert *et al.*^18^ recently demonstrated that although DBA1/J mice immunized using citrullinated fibrinogen apparently produced ACPA, they failed to develop clinical arthritis. Together with our data, it can be hypothesized that the presence of ACPA is insufficient for arthritis development; rather, ACPA appears to be able to enhance arthritis triggered by anti-CII antibodies.

Although several lines of evidence support the involvement of ACPA in the pathogenetic mechanism of RA, the contribution of ACPA to the development or deterioration of the disease remains unclear. Sokolove *et al.*^23^ suggested that immune complexes containing ACPA and citrullinated fibrinogen stimulated macrophages via Toll-like receptor-4 and Fcγ receptor. Harre *et al.*^24^ more recently demonstrated that antibodies to citrullinated vimentin induced osteoclasts that contributed to bone erosion in RA. The elucidation of the precise pathogenetic mechanism of ACPA is a very important and interesting topic for future research. We observed that elimination of ACPA at the preclinical stage, when overt arthritis was not evident, could prevent arthritis development. ACPA can be present many years before the onset of clinical arthritis in human RA.^3, 16^ Therefore, the question arises as to whether the development of overt arthritis can be prevented at the preclinical stage in the ACPA-positive population. Application of our finding to human RA would provide a new approach to preclinical arthritis management. However, there are obstacles that need to be addressed in terms of the technique to selectively target ACPA-producing B cells and to suppress peptidyl arginine deiminase to an optimal level. In addition, it would be interesting to investigate the effect of ACPA elimination following establishment of arthritis.

In conclusion, we developed a novel ACPA, 12G1, that identified a range of citrullinated proteins in arthritic joints in various experimental arthritis models. We also demonstrated that 12G1 had arthritogenic potential in CIA mice. Targeting ACPA-producing B cells or suppressing peptidyl arginine deiminase to an optimal level could be a promising approach to RA treatment.

## METHODS

### Animals

To generate 12G1 antibody, 6-week-old female BALB/c mice (Harbin, China) were used. For CIA and CAIA, 6−8-week-old male DBA1/J mice were purchased from OrientBio (Seongnam, Korea). IL-1Ra KO mice were kindly provided by Professor Yoichiro Iwakura (Center for Experimental Medicine, Institute of Medical Science, University of Tokyo, Tokyo, Japan). The experimental mice were housed in filter-top cages, with water and food provided *ad libitum*. All animal research procedures were performed in accordance with the Laboratory Animals Welfare Act, the Guide for the Care and Use of Laboratory Animals and the Guidelines and Policies for Rodent Experiment provided by the Institutional Animal Care and Use Committee in the School of Medicine, The Catholic University of Korea (CUMC-2011-0062-01).

### Immunization of mice and preparation of the monoclonal antibody

A citrullinated peptide (CCP: HQCHQESTXGRSRGRCGRSGS; X=citrulline) and a noncitrullinated peptide (CRP: HQCHQESTRGRSRGRCGRSGS) were synthesized. The CCP synthetic peptide was intraperitoneally injected into four 6-week-old female BALB/c mice in conjunction with complete Freund's adjuvant (Chondrex, Redmond, WA, USA) to generate antibody to the CCP peptide. As a booster, the mice were injected using CCP diluted in phosphate-buffered saline (PBS) 4 and 8 weeks after the first immunization. After 3 days, B cells were isolated from the mouse that demonstrated the highest binding affinity for CCP via serum ELISA (method described below) and fused with myeloma cells (Sp2/0-Ag14) using polyethylene glycol (10 783 641001, Roche, Branchburg, NJ, USA).

The fused cells were cultured in 1 × hypoxanthine-aminopterin-thymidine culture medium (H0262, Sigma, St Louis, MO, USA). Cells displaying a positive ELISA test signal (see below) were transferred to a 24-well plate. After separating the cells into 96-well plates, they were cultured for 7–10 days in hypoxanthine and thymidine culture medium (11067-030, Gibco, Carlsbad, CA, USA) under 5% CO_2_ at 37 °C in an incubator. Hybridoma cells were screened using ELISA (see below) and the cloning process was repeated until the final antibody secreting clone was selected.

### ELISA for antibody screening

CCP or CRP (negative control) were diluted (to 5 μg ml^−1^) in coating buffer and 50 μl were coated onto each well of an ELISA plate either overnight at 4 °C or for 2 h at 37 °C. The plates were blocked using 2% skimmed milk/Tris-buffered saline with Tween-20 at 37 °C for 1 h. Sera from immunized mice, or the supernatant from the hybridoma cells, were loaded into the wells and incubated for 2 h at room temperature. The plates were washed and 50 μl of horseradish peroxidase-conjugated goat anti-rabbit immunoglobulin G (IgG) (31439, Pierce, Rockford, IL, USA; 1:5000 in blocking buffer) was added to each well for 1 h at room temperature. Finally, 50 μl of chromogenic substrate (3,3′,5,5′-tetramethylbenzidine; SurModics, Eden Prairie, MN, USA) was added to each well for 30 min, followed by 50 μl of stop solution (1 N H_2_SO_4_). The absorbance at 450 nm was read in an ELISA plate reader. In addition, serum samples were collected from mice 4 weeks after the primary immunization and tested as described above.

### Induction of CIA and CAIA

To induce CIA, 6-week-old male DBA1/J mice (OrientBio) were immunized intradermally into the base of the tail using bovine CII (100 μg per mouse; Chondrex) emulsified in Freund's complete adjuvant (Arthrogen-CIA, Chondrex). After 2 weeks, 100 μg CII dissolved and emulsified at the same concentration using incomplete Freund's adjuvant (Difco, Detroit, MI, USA) was administered intradermally as a booster injection. To induce CAIA, anti-CII antibody (2 mg; Chondrex) was injected intravenously into 6-week-old male DBA1/J mice (OrientBio), and 3 days later, lipopolysaccharide (50 μg) was administered intraperitoneally.

### Induction of 12G1 antibody-induced arthritis

Male 6-week-old DBA1/J mice (OrientBio) were immunized intradermally into the base of the tail using bovine CII (100 μg per mouse); Chondrex) emulsified in Freund's complete adjuvant (Arthrogen-CIA, Chondrex). After 3 weeks, the mice were injected intraperitoneally using 1.5 mg 12G1 antibody or isotype IgG in 200 μl PBS instead of conventional second boosting. After 7 days, the same concentration of 12G1 antibody or IgG was injected intraperitoneally. As a negative control, 12G1 antibody was incubated with beads (protein A/G plus-Agarose, SC-2003, Santa Cruz Biotechnology, Dallas, TX, USA) for 4 h at 4 °C. After centrifugation (250 *g*, 30 s), only the supernatants were collected that were injected into the mice according to the method used for the 12G1 antibody.

### Assessment of arthritis severity

Arthritis severity was determined by two independent observers. The mice were examined twice weekly (CIA) or daily (CAIA) for the onset and severity of joint inflammation for up to 7 weeks after primary immunization. The arthritis severity was assessed on a scale of 0–4, using the following criteria: 0=no evidence of erythema or swelling; 1=erythema and mild swelling confined to the mid-foot (tarsals) or ankle joint; 2=erythema and mild swelling extending from the ankle to the mid-foot; 3=erythema and moderate swelling extending from the ankle to the metatarsal joints; and 4=erythema and severe swelling encompass the ankle, foot and digits. The arthritic score for each mouse was expressed as the sum of the scores for all four limbs. Therefore, the maximum arthritis score for a mouse was 16. The mean arthritis index was used to compare the data among the control and experimental groups.

### Histology

Each hind paw was fixed in 4% paraformaldehyde, decalcified in ethylenediaminetetraacetic acid bone decalcifier, embedded in paraffin and sectioned (4 μm thick). The slide-mounted sections were deparaffinized in xylene and rehydrated through a series of diluted alcohols (from 100 to 70%), finally rinsing in tap water. To block endogenous peroxidase activity, the sections were incubated with 3% hydrogen peroxide for 15 min. The slides were washed three times with PBS for 5 min at room temperature and the sections stained using hematoxylin and eosin. To detect cartilage, the sections were stained using Safranin O or Toluidine Blue. Stained sections were evaluated for the presence of hyperplasia of the synovial membrane, pannus formation, cartilage erosion and infiltration by three blinded independent observers and graded using a semiquantitative score modified according to Wruck *et al.*^25^

### Immunohistochemistry

Each hind paw was embedded in paraffin, sectioned (4 μm thick), mounted on silanized charged slides and baked at 60 °C for 60 min. The mounted sections were deparaffinized, rehydrated and endogenous peroxidase activity blocked as for histology. The sections were washed with tap water and blocked using 10% normal horse serum in 1% bovine serum albumin in PBS. After washing with 0.1% Tween-20 in PBS, the sections were incubated with 12G1 (1:50) overnight at 4 °C. A biotinylated horse anti-mouse IgG secondary antibody (1:200) was applied for 40 min at room temperature followed by the RTU VECTASTAIN Elite ABC reagent (PK-6102; Vector Laboratories, Burlingame, CA, USA) for 10 min. Tissue staining was visualized after incubation with the 3,3′-diaminobenzine substrate chromogen solution (Sk-4100, Vector Laboratories) for 1.5 min. The sections were counterstained for 1 min using Mayer's hematoxylin (131-09665, Wako, Richmond, VA, USA), dehydrated and mounted. The isotype (negative) control was a mouse IgG1 monoclonal antibody (5415S, Cell Signaling Technology, Danvers, MA, USA). The sections were visualized using the LEICA SCN 400 imaging system (Leica microsystems, Buffalo Grove, IL, USA), and the areas with positive cells were calculated utilizing the Visiopharm integrator system version 4.3 (Visopharm, Horsholm, Denmark).

### Immunofluorescence

Slide-mounted sections of each mouse model were deparaffinized in xylene and rehydrated using a graded series of ethanol. The sections were treated using citrate buffer for antigen retrieval and blocked using 10% normal goat serum. The localization of each protein was probed using the relevant primary antibody: polyclonal anti-vimentin (B2312; Santa Cruz Biotechnology), anti-fibronectin (ab23750; Abcam, Cambridge, MA, USA), anti-collagen (ab34712; Abcam) and anti-BiP (ab53068; Abcam) antibody (diluted 1:100 in assay buffer) according to the manufacturers' instructions. Simultaneously, sections were incubated with 12G1 antibody (1:50). The secondary antibodies Alexa488-anti-mouse and Alexa594-anti-rabbit (Thermo Fisher, Rockford, IL, USA) were used to label citrullination and target proteins, respectively. Protein expression was quantified based on staining intensity. Immunofluorescence photomicrographs were acquired using a Leitz Diaplan microscope (Leica) coupled to the Leica/Wild MPS48 automated photographic system.

### Statistical analysis

Statistical analyses were performed using the GraphPad Prism 4.0 software (GraphPad, San Diego, CA, USA) and data are presented as mean±s.d. Data were compared using two-factor analysis of variance with Bonferroni's post-test or the Mann–Whitney *U-*test. A value of *P*<0.05 was taken to indicate statistical significance.
